# The Effect of Comprehensive Care on the Patients Received Minimally Invasive Percutaneous Nephrolithotomy

**Published:** 2017-07

**Authors:** Xue-Li WEI, Mei-Fang XUE, Zhao-Xia QIN, Xing-Yun BAI, Fang-Fang DONG, Jin-Jin ZHANG, Ning LV, Hui CHEN, Jia ZHANG

**Affiliations:** 1. Dept. of Urinary Surgery No.2, Zhumadian Central Hospital, Zhumadian, China; 2. Physical Examination Center, Zhumadian Central Hospital, Zhumadian, China

**Keywords:** Minimally invasive percutaneous nephrolithotomy, MPCNL, Renal lithiasis

## Abstract

**Background:**

We analyzed the effect of comprehensive care on the patients who received minimally invasive percutaneous nephrolithotomy (MPCNL).

**Methods:**

Patients hospitalized from 2013–2014 in Zhumadian Central Hospital (n=124) were enrolled and divided into two groups on random basis. The control group was treated with routine nursing model while the observation group was given comprehensive care additionally. The surgery time, degree of comfort, complications and successful cases, hospitalization time, sleep quality, nursing satisfaction and changes of systolic pressure, pulse and respiratory at different time were observed and analyzed.

**Results:**

The surgery time of the control group was significantly longer than that of observation group (*P*<0.05). The observation group felt more comfortable and showed more significant successful cases than the control group. Moreover, the hospitalization time were significantly reduced in observation group when compared with control group (*P*<0.05). The sleep quality of the observation group was significantly better than that of the control group (*P*<0.05). Before anesthesia, diastolic blood pressure, systolic blood pressure, pulse and respiration were not significantly different between the two groups. The diastolic blood pressure, systolic blood pressure, pulse and respiration after anesthesia, intraoperative 30 min, postoperative 30 min and other moments were significantly different. The incidence of complications in the control group was significantly higher than that in the observation group. The nursing satisfaction of the observation group was significantly higher than that of the control group.

**Conclusion:**

The comprehensive care on the patients undergoing MPCNL was effective, and it can dramatically shorten surgery time, improve the success rate, improve the sleep quality of patients, keep life sign stable and minimize the complications.

## Introduction

Minimally invasive percutaneous nephrolithotomy (MPCNL) is the main treatment of urinary calculi. The MPCNL uses X-ray or ultrasound guidance; get the stone under the direct vision with ureterorenoscopy to remove urinary tract obstruction, in order to achieve the purpose of treatment (1). Because it has the advantages of less trauma, fewer complications, faster post-operative recovery, and shorter hospital stay, it gradually replaced the traditional open stone surgery (2). In addition, MPCNL also has a high stone clearance rate. Moreover, for residual stones and recurrent stones it can give the stone twice, recognized by the majority of patients and medical personnel (3–4). In spite of this, the difficulty of surgery is still large, requiring patients with high degree of cooperation, perioperative nursing intervention is also very important for the prognosis (4–5).

In this study, we evaluated the comprehensive nursing intervention effect on the patients with MPCNL for the treatment of kidney stones.

## Methods

### Patients

Total of 124 patients were selected from the Department of Urology in Zhumadian Central Hospital, HeNan, China between June 2013 and December 2014. Among them, 90 men and 34 women (average age 50.2 ± 7.5 yr old) were included with minimum 37 years age and maximum 65 years of age. For the diagnosis of kidney stones, the patients were diagnosed by intravenous or ultrasound, and confirmed by CT if necessary. There were MPCNL indications. All cases were randomly divided into observation group and control group, with 62 cases in each group. There was no significant difference in gender, age and culture between the two groups and they were comparable (*P*>0.05).

This study was approved by the Ethics Committee of Zhumadian Central Hospital. Signed written informed consents were obtained from the patients and/or guardians.

### Operation method

After the success of the continuous epidural anesthesia, the patients were asked for lithotomy position, and then the ureteral catheter was inserted into the affected side. After its success, the body was turned to the prone position. Under ultrasound localization, in the lower edge of the rib 12, the renal pelvis was punctured with No.18 puncture needle. Further, under the guidance of zebra guide wire, the nephroscope was inserted to take stone. The procedure requires proper amount of contrast agent through the ureter, position under C arm X-ray machine monitoring, with the direction of puncture vertically in the renal parenchyma. Also it requires to establish percutaneous renal access, crush stones by holmium laser and lavage round-trip by infusion pump high pressure pulse, to take large stones with lithotomy forceps.

### Nursing methods

#### Common for two groups

The two groups were given routine nursing care, humanistic care and health education. The nursing staff kept the operation room quiet and clean. After sending the patient to the operation room, nurses encouraged to bring comfort for patients, and cooperated with the doctors for patients placed in the right position. The nurses monitored the changes of blood pressure, heart rate, breathing and other vital signs of the patients during the operation, and report the abnormal situation to the doctor in time. After the success of anesthesia in patients, the nurse assisted the patient to take the position of the stone to meet the need of the operation. After the reteral catheter placement, the nursing staff put down the double lower limbs of the patient and assisted him to change to the supine position, let his hands hold the head, and the nurses disaggre-gated on both sides of the patients, and assisted his reversing rolling into a prone position. The nurses help to roll into a flip position after the end of surgery. It is required for nurses to pay attention to changing body position and not let the pipe to come off, and the operation room temperature should be controlled 22–24 °C.

### Comprehensive nursing intervention

1) Preoperative nursing: The patient was explained for the characteristics and process of the operation, the need to change the body position if necessary. The patients were allowed to watch first surgery video, and allowed to carry out the posture exercises with the guidance of the nurses. The exercise started from 30min and gradually extended to 1 h, 2, 3h. The patients can remain lithotomy position for 30 minutes and stay in the prone position for 3 hours. During the course, no abnormal signs of life mean the success of practice and training.

2) Intraoperative nursing: First of all, the nurses were trained to understand the responsibilities of the operation and to cooperate with the operation room nursing (6). [1] Body position nursing during operation was the same as above; [2] Psychological nursing during operation: If there was anxiety, tension and fear, nursing staff used humor language, smile to comfort the patients or to guide patients to relax such as deep breathing, or play music; [3] Intraoperative temperature nursing: In order to avoid the cold stimulation, in addition to adjusting the room temperature to the most suitable scope, attention was given to cover the warmth. The liquid was warmed with a constant temperature chamber and kept closer to the body’s 35–37°C; [4] Intraoperative monitoring of life sign: close attention was given to the heart rate, blood pressure, respiration etc., because intraoperative postural changes, traction of operation and too long fixed position time will affect the patient’s mood, blood pressure, heart rate and respiration. Therefore it is necessary to pay close attention, if there is abnormal, it needs timely treatment.

### Observation index

The operation time, hospitalization time, number of cases which were operated successfully, complication, comfort (investigation of patients, with the option of comfortable or not comfortable) of two groups were compared. Further, the sleep quality, systolic pressure variation at the different time, variation of pulse and breath between the two groups after treatment, the patient’s sleep quality and nursing satisfaction were compared.

Patients’ sleep quality was evaluated according to the subjective experience of the patients: [1] Good sleep: The patients sleep faster, feeling deep sleep, sleep time is over 7 h, after waking up feeling state of mind is good; [2] Poor sleep: Patients sleep relatively slow, feeling shallow sleep, sleep time is less than 7 hours, after waking up, and patients are in poor mental state and feel fatigue.

### Processing method

The data were analyzed by SPSS 18.0 statistical software, measurement data were expressed by x±S with t test. The data was tested by chi square, and was expressed as percentage. The difference with *P*<0.05 was taken as statistical significant difference.

## Result

### Operation time, comfort and operation success of two groups

The operation time of the control group was significantly longer than the observation group (*P*< 0.05). The hospitalization time of observation group was significantly lower than that of control group (*P*< 0.05). The operation success of control group 56 was significantly lower than the observation group 60 (*P*< 0.05). It can be seen that the comprehensive nursing intervention can significantly reduce the operation time, improve the comfort and the success rate of surgery (Table 1).

**Table 1:** Comparison of operation time, comfort and operation success of two groups

**Group**	**Number of cases**	**Operation time (x̄±S) min**	**Hospitalization time (x̄±S) d**	**Operation success [n (%)]**
Control	62	134.5±11.9	12.6±3.2	56(90.3)
Observation	62	118.2±9.5^*^	7.4±1.7^*^	60 (96.8)^*^

Note: Compared with the control group,

* *P* < 0.05

## Comparison of sleep quality and comfort between two groups of patients after treatment

For the sleep quality comparison after treatment, in the control group 32 (51.6%) patients slept well, in the observation group 54 (87.1%) patients slept well, the difference between the two groups was significant (*P*<0.05). For the comfort comparison after treatment, in the control group 48 patients (77.4%) felt comfortable, in the observation group 58 (93.5%) patients felt comfortable, the difference between the two groups was significant (*P*<0.05), as shown in Table 2.

**Table 2:** Comparison of sleep quality and comfort between two groups of patients after treatment

**Group**	**Number of cases**	**Sleep quality [n (%)]**		**Comfort [n (%)]**	
		Good sleep	Poor sleep	Comfortable	Discomfortable
Control	62	32(51.6)	30(48.4)	48(77.4)	14(22.6)
Observation	62	54(87.1)^*^	8(12.9)^*^	58(93.5)^*^	4(6.5)^*^

Note: compared with the control group,

* *P<*0.05

### Changes of diastolic blood pressure in two groups

**Fig. 1:**
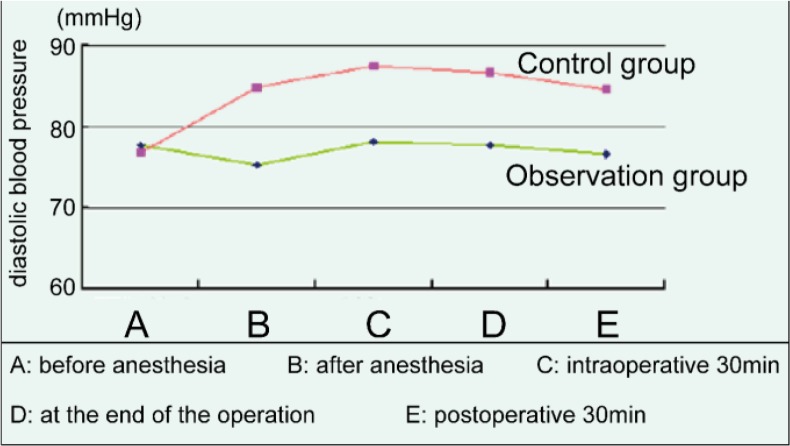
Changes of diastolic blood pressure in two groups

Before anesthesia, there was no significant difference in diastolic blood pressure between the two groups. There were significant differences in diastolic blood pressure between the two groups after anesthesia, intraoperative 30min, at the end of the operation, and postoperative 30min (*P*<0.05), among which the diastolic blood pressure in the observation group was more stable, as shown in Fig. 1.

### Changes of systolic blood pressure in two groups of patients at different times

**Fig. 2:**
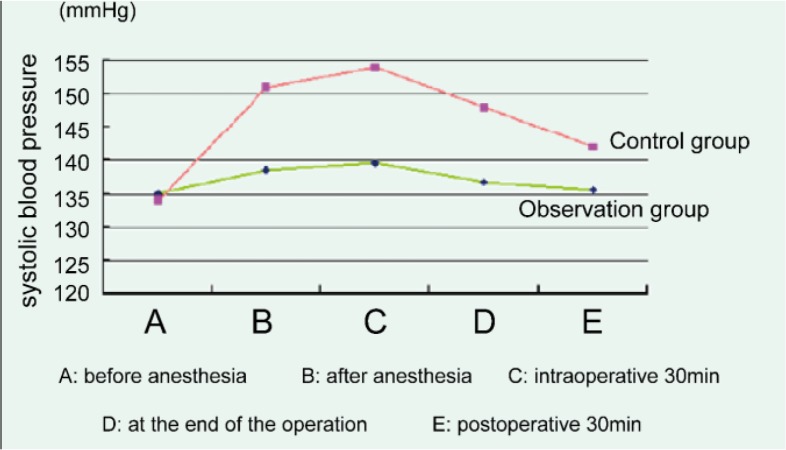
Changes of systolic blood pressure in two groups of patients at different times

Before anesthesia, there was no significant difference in systolic blood pressure between the two groups. There were significant differences in systolic blood pressure between the two groups after anesthesia, intraoperative 30min, at the end of the operation, and postoperative 30min (*P*=0.017), among which the systolic blood pressure change in the observation group was more stable, as shown in Fig. 2.

### Pulse changes in two groups of patients at different time

**Fig. 3:**
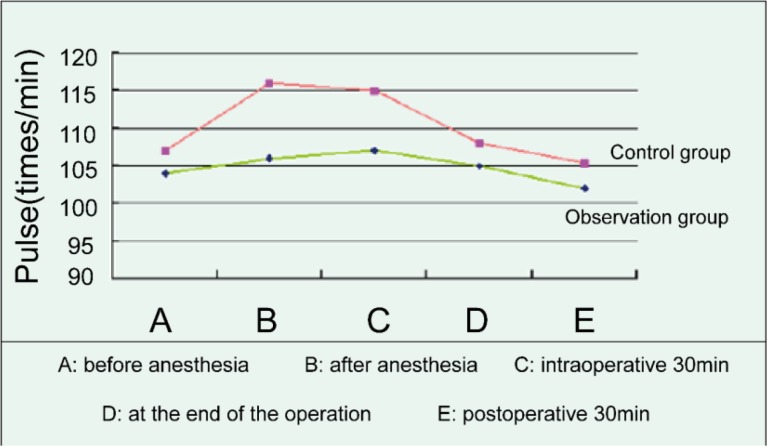
Pulse changes in two groups of patients at different time

Before anesthesia, there was no significant difference in pulse changes between the two groups. There were significant differences in the pulse comparison between the two groups after anesthesia, intraoperative 30 min, at the end of the operation, and postoperative 30min (*P*=0.026), among which the pulse rate of the control group was higher than that of the observation group, while the observation group was relatively stable, as shown in Fig. 3.

### Respiratory changes of two groups of patients at different time

**Fig. 4:**
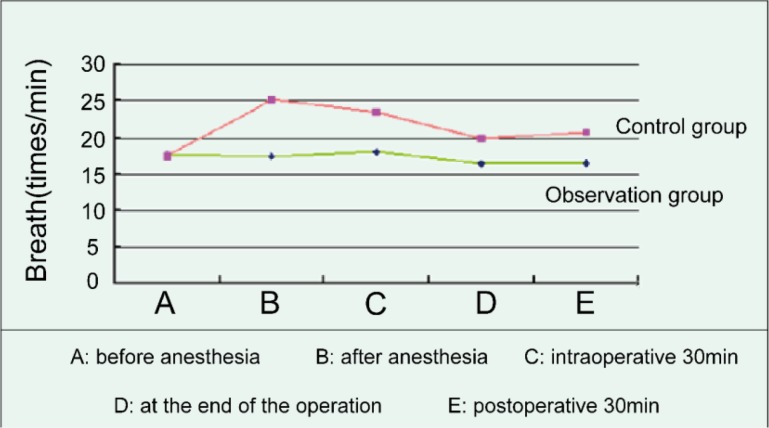
Respiratory changes of two groups of patients at different time

Before anesthesia, there was no significant difference in respiratory between the two groups. There were significant differences in the respiratory comparison between the two groups after anesthesia, intraoperative 30min, at the end of the operation, and postoperative 30min (*P*=0.037), among which, the respiratory rate in the control group was higher than that of the observation group, while the observation group was relatively stable, as shown in Fig. 4.

### Comparison of complications of two groups of patients

There was a total of 4 people with complications in the observation group (6.5%), less than 9 people in the control group of (14.5%) with statistically significant difference (*P*=0.019). It indicated that comprehensive nursing intervention could significantly improve the prognosis.

### Comparison of nursing satisfaction between the two groups

The nursing satisfaction of the observation group was 87.1% (54/62 cases) was significantly higher than the nursing satisfaction of the control 77.4% (48/62 cases) (*P*=0.035).

## Discussion

Kidney stone is a common disease in the department of urology. Minimally invasive percutaneous nephrolithotomy has the advantages of less trauma, faster recovery etc., (7–11) which is gradually replacing the traditional open surgery to become the main method for the treatment of the disease. However, there is still need to pay attention to the cooperation of the operation including patients, nurses etc. (12). In particular, the nursing intervention is very important. Under the careful nursing, it can significantly improve patients’ comfort degree, the success rate of the operation, and shorten the operation time (13–15). The psychological nursing in operation allows the patient to relax with not large swings in mood, because many patients do not understand the operation process and get worried about the pain and surgical effect operation brings. In that case, the medical staff needs to explain to the patient, or let the patient observe surgery video, understand the prognosis and the importance of the coordination of the operation as well as the advantages of the operation. At the time of surgery, the nurse can talk with the patient, or play music to distract attention. In addition, washing the stone in the surgery will take away a lot of temperature, so heating fluid to maintain the body temperature of patients is also very important, it will make patients feel more comfortable in the surgery (16–17).

The study showed that the operation time of patients in the control group was significantly longer than those of the observation group. The comfort and success rate of the control group was significantly lower than that of the observation group (<0.05). The hospitalization time of observation group was significantly lower than that of control group (*P*<0.05). The sleep quality of the observation group was significantly better than that of the control group (*P*<0.05). It suggested that comprehensive nursing intervention could improve patients’ comfort and sleep quality, and reduces the operation time and hospitalization time of patients.

The change of vital signs in patients is also a problem that must be paid attention to in the surgical operation. (18) This study showed that before anesthesia, there was no significant difference in diastolic blood pressure, systolic pressure, pulse, respiration between the two groups (*P*>0.05). The diastolic and systolic blood pressure, pulse, respiration after anesthesia, intra-operative 30min, at the end of the operation, and postoperative 30min of two groups had significant difference (*P*<0.05), among which the fluctuation range of the control group was higher than that of the observation group, while the observation group was relatively stable. The research suggested that the comprehensive nursing intervention is beneficial to the stability of the vital signs of the patients during the operation and after the operation.

Nursing satisfaction is an important index to measure the quality of nursing care for patients with nursing intervention. Low nursing satisfaction showed that the patients had lower degree of recognition of nursing service, and the relationship between nurses and patients was poor. This study showed that nursing satisfaction of the observation group was 87.1%, significantly higher than that of the control group 77.4% (*P*<0.05), which concurs with earlier reports (19). At the same time, careful nursing is a key to the success of surgery as wells as reducing the complications. In this study, the complications of the observation group was significantly lower than that of the control group (*P*<0.05), as reported earlier (20).

## Conclusion

The implementation of comprehensive nursing intervention in minimally invasive percutaneous nephrolithotomy for the treatment of renal calculi better maintains the stable vital signs of the patients with less postoperative complications, and the nursing intervention is effective, that is worth popularization and application.

## Ethical considerations

Ethical issues (Including plagiarism, informed consent, misconduct, data fabrication and/or falsification, double publication and/or submission, redundancy, etc.) have been completely observed by the authors.
